# New Insights of Cardiac Arrhythmias Associated with Sleep-Disordered Breathing: From Mechanisms to Clinical Implications—A Narrative Review

**DOI:** 10.3390/jcm14061922

**Published:** 2025-03-12

**Authors:** Mariela Romina Birză, Alina Gabriela Negru, Ștefan Marian Frent, Andreea-Roxana Florescu, Alina Mirela Popa, Andrei Raul Manzur, Ana Lascu, Stefan Mihaicuța

**Affiliations:** 1 Center for Research and Innovation in Precision Medicine of Respiratory Diseases, Department of Pulmonology, “Victor Babes” University of Medicine and Pharmacy Timisoara, Eftimie Murgu Sq. No. 2, 300041 Timisoara, Romania; romina.birza@umft.ro (M.R.B.); frentz.stefan@umft.ro (Ș.M.F.); andreea.florescu@umft.ro (A.-R.F.); alina-mirela.popa@umft.ro (A.M.P.); andrei.manzur@umft.ro (A.R.M.); stefan.mihaicuta@umft.ro (S.M.); 2 Department of Cardiology, “Victor Babes” University of Medicine and Pharmacy, 300041 Timisoara, Romania; 3 Discipline of Pathophysiology, Department III of Functional Sciences, “Victor Babes” University of Medicine and Pharmacy, Splaiul Tudor Vladimirescu nr. 14, 300173 Timisoara, Romania; lascu.ana@umft.ro

**Keywords:** arrhythmia, obstructive sleep apnea, atrial fibrillation, sudden cardiac death, screening, CPAP, pharmacotherapy, atrial fibrillation ablation, OSA related mechanisms

## Abstract

Although most research has concentrated on the link between sleep apnea and atrial fibrillation, obstructive sleep apnea (OSA) is also associated with ventricular arrhythmias. These cardiac arrhythmias can be triggered by repeated episodes of hypoxemia, hypercapnia, acidosis, intrathoracic pressure fluctuations, reoxygenation, and other mechanisms that occur during apnea and hypopnea. Studies show that OSA reduces the effectiveness of arrhythmia treatments, such as antiarrhythmic medications and radiofrequency current ablation. Several non-randomized studies indicate that treating sleep apnea syndrome with continuous positive airway pressure (CPAP) may help maintain sinus rhythm following electrical cardioversion and increase the success rates of catheter ablation. This review aims to thoroughly examine the role of OSA in the development of cardiac arrhythmias. Screening for OSA and arrhythmias in patients with OSA provides vital information on the need for additional interventions, such as CPAP therapy, anticoagulation, antiarrhythmic drug therapy, catheter ablation for specific arrhythmias, or device therapy. New therapies for OSA treatment have the potential to significantly influence arrhythmia development in patients with sleep-disordered breathing. However, further research is required to validate these findings and formulate comprehensive treatment protocols.

## 1. Introduction

Sleep apnea syndrome is a prevalent condition marked by recurrent episodes of total (apnea) and partial (hypopnea) blockage of the upper airway during sleep, causing shallow breathing. Apnea is defined as a reduction in nasal airflow by at least 80% for a minimum duration of 10 s, while hypopnea is characterized by a decrease in nasal airflow of 30% or more, accompanied by a reduction in oxygen saturation of at least 4% [1]. The most common breathing irregularity associated with sleep is obstructive sleep apnea (OSA). This occurs due to the altered airway muscle tone and tongue, leading to the obstruction of the upper airways. Sleep apnea syndrome has been linked to various cardiac arrhythmias. While most studies have focused on the relationship between sleep apnea and atrial fibrillation (AF), there are multiple associations of OSA with ventricular arrhythmias. Cardiac arrhythmias can be provoked by recurring episodes of hypoxemia, hypercapnia, acidosis, fluctuations in intrathoracic pressure, reoxygenation, and awakenings that occur during apnea and hypopnea [2]. Various altered electrophysiological parameters, including abnormal automaticity, triggered automaticity, atrial ERP shortening, QT interval prolongation, and re-entry mechanisms, may be induced by the pathophysiology of sleep apnea syndrome. Apneas and hypopneas affect gas exchange and lead to oxygen desaturation, especially in people with underlying pulmonary or cardiovascular disease. The cessation of upper airway obstruction following a respiratory event can lead to reoxygenation and dangerous reactive oxygen species (ROS) formation. Oxidative stress is implicated in myocardial hypertrophy, injury and apoptosis, leading to structural cardiac modifications in animal models. The generation of ROS has been associated with arrhythmogenesis in both animals and humans due to alterations in calcium channel activity and inducing microvascular ischemia. Hypoxemia leads to peripheral vasoconstriction, thereby causing both increased preload and afterload. Oxidative stress favors fibroblast activation in myofibroblasts, leading to perivascular and interstitial fibrosis, subsequently causing slowed and heterogeneous conduction. Echocardiographic evidence of inter- and intra-atrial electromechanical delay and P-wave dispersion on the ECG was observed in moderate to severe sleep apnea syndrome. Furthermore, left ventricular hypertrophy, which predisposes to ventricular arrhythmias and conduction delay, was observed to be particularly pronounced in individuals with both sleep apnea and hypertension. In conclusion, sleep apnea syndrome is associated with atrial remodeling, dilatation, and left ventricular hypertrophy, leading to electrophysiological alterations predisposing to arrhythmogenesis.

Research indicates that OSA diminishes the effectiveness of arrhythmia therapies, including antiarrhythmic medication and radiofrequency current ablation. Several non-randomized studies suggest that treating sleep apnea syndrome with continuous positive airway pressure (CPAP) may aid in maintaining sinus rhythm after electrical cardioversion and enhance the success rates of catheter ablation [3]. This review aims to offer a close examination of OSA in the genesis of cardiac arrhythmias.

## 2. Epidemiology and Pathophysiology

OSA is characterized by an apnea-hypopnea index (AHI) exceeding 10 events per hour. A normal AHI is considered to be less than 5 events per hour, while severe apnea is characterized by an AHI of over 30 events per hour [4,5]. OSA is particularly prevalent, with an estimated 1 billion adults affected worldwide. Despite being widely prevalent, OSA continues to be largely undiagnosed, particularly among racial and ethnic groups that have experienced historical and systemic poor socio-economic environments [6,7].

The under-representation of black individuals in OSA research was discussed by Olafiranye O et al., particularly concerning the relationship between OSA and CV disease, with significant public health implications, given the disproportionately high rates of sudden cardiac death among these populations. There is limited evidence linking OSA-related arrhythmias to adverse CV events in black individuals. The few large-scale studies that have included substantial numbers of black participants, such as the Jackson Heart Study, were also mentioned. However, these studies often rely on self-reported sleep symptoms rather than objective evaluations like polysomnography [8]. In another study, black men experienced the most severe OSA, exhibiting the highest AHI, greatest sleep fragmentation, and most severe hypoxemia. Black women were also more likely to be diagnosed with severe OSA compared to White women. Several factors contributing to this delay include beliefs that snoring is normal, attributing OSA symptoms to age-related insomnia, concerns about sleep laboratory testing, and systemic barriers such as access to nighttime testing, childcare, and time off from work [9].

Individuals with possible undiagnosed (versus diagnosed/treated) OSA had greater odds of being a minoritized race Hispanic/Latino, uninsured, and were less likely to have more education, high income, and be women [10]. 

In a working population aged 30–39 years, the prevalence of OSA was 5% in females and 12% in males. Recent research showed an even higher prevalence of sleep apnea in young adults, with 16% being affected. Moreover, in individuals over 65 years of age, the prevalence of sleep apnea is at least double, ranging from 13% to 39% [11,12]. The immediate consequences of sleep-disordered breathing (SDB) encompass intrathoracic pressure changes (specific to OSA), repetitive episodes of hypoxia, hypercapnia, reoxygenation, autonomic system activity fluctuations, and disturbances in sleep architecture.

**Figure 1:**
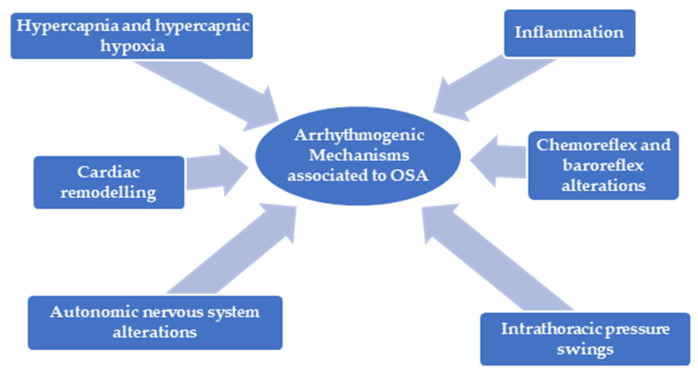
A mechanism-based approach to OSA—associated arrhythmogenesis. OSA—obstructive sleep apnea.

The constant physiological stress induced by sleep-disordered breathing results in sustained biological effects, leading to cardiovascular substrate alterations and an increased risk for cardiac arrhythmogenesis [6]. The foundation of arrhythmogenesis in OSA encompasses changes in cardiac automaticity, triggered activity and re-entrant mechanisms induced by major factors as autonomic nervous system alteration, hypercapnia, hypercapnic hypoxia, chemoreflex and baroreflex alterations, intrathoracic pressure variations and inflammation (Figure 1).

### 2.1. Autonomic Nervous System Alterations

OSA-induced alterations in the autonomic nervous system bidirectionally modulate arrhythmogenesis, implicating both sympathetic and parasympathetic overactivity. In relation to AF, both sympathetic and parasympathetic activation contribute to arrhythmogenesis. However, parasympathetic activation exerts a protective effect in the context of ventricular arrhythmias, whereas sympathetic activation promotes the occurrence of these arrhythmias [13,14]. Sympathetic activation predominantly occurs during apnea episodes due to hypoxia and sleep arousal, consequent to breathing difficulty [15,16]. Sleep fragmentation contributes to increased cardiovascular risk and exacerbates sympathetic activation, thereby disrupting the balance between the sympathetic and parasympathetic systems [16]. Another trigger for sympathetic activation is the cyclic hypoxia-reoxygenation process, which activates multiple molecular pathways that are also involved in inflammation, procoagulability, and endothelial dysfunction [17].

### 2.2. Hypercapnia and Hypercapnic Hypoxia

Another primary mechanism involved in arrhythmogenesis is the episodic occurrence of intermittent hypoxia and elevated carbon dioxide levels, which induce a shortening of the effective myocardial refractory period (ERP), thereby increasing electric vulnerability. The recurring pattern of hypoxia followed by reoxygenation has been shown to activate multiple cellular and molecular pathways, like inflammation and oxidative stress [18]. Hypercapnia induces sympathetic overstimulation, affecting peripheral blood vessels, a response that may be modulated by hyperventilation. The simultaneous occurrence of hypercapnia and hypoxemia can lead to an amplified sympathetic response [19]. On the other hand, recurrent hypoxemia results in elevated levels of ROS, which influence cardiac automaticity by disrupting potassium ion regulation [20].

### 2.3. Chemoreflex and Baroreflex

The chemoreflex is defined as the feedback system that modulates ventilation in response to variations in blood oxygen and carbon dioxide levels. OSA appears to be associated with hypersensitivity of the chemoreflex and overstimulation of the baroreflex in response to carotid sinus sensitivity to hypoxia, resulting in prolonged sympathetic activation [21]. Hypercapnia simultaneously affects the carotid body receptors and the medulla oblongata chemoreceptors. Current data suggest that peripheral chemoreceptors can enhance the sensitivity of central chemoreceptors to hypercapnia over afferent innervation [22]. Interesting data from a rodent study indicate that chronic intermittent hypoxia in rats leads to the development of new noradrenergic terminations within various sensory and motor regions of the lower brainstem. This finding suggests that patients with OSA may exhibit similar adaptive increases in airway reactivity. Chronic intermittent hypoxia could enhance chemoreflex responses, potentially inducing sympathetic activation [23].

### 2.4. Intrathoracic Pressure Swings

When breathing against a collapsed upper airway, negative intrathoracic pressure is generated. This negative pressure increases the collapsibility of the surrounding soft tissues, exacerbating airway obstruction. During inspiration, as the diaphragm contracts and the chest wall expands, a vacuum effect is created, which pulls in collapsed or lax soft tissues, leading to airway obstruction. This phenomenon is notably pronounced during obstructive events in OSA, worsening respiratory disturbances’ severity [15,24]. On the other hand, alterations in intrathoracic pressure result in intracardiac transmural pressure gradients and myocardial stretching, further intensifying sympathetic stimulation [6,25]. Recent research revealed that of patients with paroxysmal AF, 56% were found to have OSA, according to sleep study results. Simulated OSA-induced intrathoracic pressure swings led to significant rates of atrial premature beats (PABs), which were important in triggering episodes of AF in the study group [26]. A recent murine model study found that simulated acute OSA by intermittent negative upper airway pressure caused a temporary increase in atrial oxidative stress. Although this stress was reversible, repeated exposure to intermittent negative upper airway pressure conditions every other day resulted in an arrhythmogenic substrate for AF. Even mild-to-moderate OSA with high night-to-night variability was found to be responsible for AF substrate progression [27].

### 2.5. Inflammation

The oxidative stress triggers an inflammatory response, releasing pro-inflammatory cytokines and chemokines. Recent research examined the relationship between hs-CRP, TNF-α, IL-6, IL-1β, and OSA. The findings demonstrated the presence of systemic inflammatory reactions in patients with OSAS. As the severity of OSAS increased, the inflammatory responses were intensified, particularly with elevated levels of serum hs-CRP [28]. OSA and intermittent hypoxia lead to the generation of reactive oxygen species (ROS) through mechanisms such as mitochondrial dysfunction, activation of NADPH oxidase and xanthine oxidase, as well as the uncoupling of nitric oxide (NO) synthase. This results in oxidative stress. The interaction between ROS and exacerbates oxidative stress, reducing NO bioavailability and contributing to endothelial dysfunction and inflammation, which are linked to hypertension, atherosclerosis, and hypercoagulability. ROS-induced sympathetic activation increases renin levels, subsequently elevating angiotensin II and endothelin 1, thereby raising blood pressure. As a signaling molecule, ROS can activate various pathways, such as mitogen-activated protein kinases and c-Jun N-terminal kinase, which then activate the nuclear factor kappa-light-chain-enhancer of activated B cells (NF-κB). With deep implications in the pathogenesis of OSA, NF-κB regulates the inflammatory response by translocating to the nucleus, increasing cytokine production from the immune cells and finally enhancing the inflammatory process [29]. Another effect of elevated ROS levels is the damage of intracellular macromolecules such as DNA, leading to cell death. These interconnected pathological processes contribute to low-grade chronic I myocardial inflammation, which is closely associated with the onset and progression of arrhythmias [30,31,32].

### 2.6. Cardiac Remodeling

The progressive cardiac remodeling concept in patients with OSA and supraventricular and ventricular arrhythmia relies on several studies that emphasize the role of patchy fibrosis in prolonging left and right atrial conduction times [33]. The most encountered remodeling mechanisms in the atria are distention with connexin alterations, consequent fibrosis, dilatation, prolonged conduction times, decreased refractoriness and high incidence of after-depolarizing potentials (ADP) [34]. On the other hand, the remodeling mechanisms in the ventricle were associated with the increased number of triggers, QT interval dispersion and structural changes such as hypertrophy and decreased left ventricular contractility leading to heart failure [35,36]. The genesis of ADPs is linked to intracellular calcium overload and was shown to arise from the atrial or ventricular hypoxic, fibrotic and electrically heterogeneous myocardial tissue [37,38]. Cardiac remodeling is also linked to episodes of sympathetic activation and blood pressure strokes during the apnea episodes, leading to transmural myocardial pressure increases [13].

## 3. Frequent Arrhythmias Associated with Obstructive Sleep Apnea

### 3.1. Atrial Fibrillation

OSA plays a role in causing, recurring, and sustaining AF. The prevalence of supraventricular (atrial and sinus) arrhythmias is estimated at approximately 50% among patients with severe OSA, compared with about 25% in patients with mild OSA and 20% in control subjects without OSA [39]. Conversely, it is estimated that between 32% and 63% of patients with AF also suffer from OSA. Furthermore, undiagnosed OSA is common among patients admitted to the hospital with AF [6,40,41]. A meta-analysis was conducted to evaluate the relationship between OSA and AF. The odds ratio for AF in OSA patients was 2.120, with a confidence interval (CI) of 1.845–2.436 and a *p*-value of <0.001, meaning that patients with OSA had more than twice the risk of developing AF compared to those without OSA [42]. The prevalence of OSA varies widely, ranging from 3% to 49% in population-based studies and from 21% to 74% in patients with AF [25]. Sleep apnea has also been linked to newly diagnosed AF following coronary artery bypass surgery and serves as an independent predictor of postoperative AF [43]. The primary mechanisms through which sleep apnea contributes to the development of AF include disturbances in the autonomic nervous system, hypoxia and fluctuations in carbon dioxide concentration, alterations in intrathoracic pressure, disruptions in circadian rhythm and nychthemeral patterns, and modifications in the cardiac substrate [6]. Sleep apnea elevates the risk of hypertension, coronary artery disease, congestive heart failure, and diabetes. Each of these pathologies, in turn, can further increase the likelihood of developing AF [44]. Current knowledge evidence indicates that autonomic nervous system disturbances are a key factor in the genesis of AF in patients with OSA. The cardiac autonomic nervous system (ANS) includes the extrinsic cardiac autonomic system, which originates from centrally derived parasympathetic and sympathetic nerves, and the intrinsic cardiac autonomic system. The latter consists of epicardial ganglionated plexi (GP) located within epicardial fat pads, which include efferent parasympathetic and sympathetic neurons, interneuronal-central nodes, afferent sensory neurons, and other cell types [45]. Previously, guidelines categorized paroxysmal AF into three types based on ANS involvement: vagally mediated, adrenergic-mediated, and mixed [46]. In a 2019 canine model study on OSA, the nerve activity before, during and after apnea, and after resiniferatoxin (RTX) injection, an analog of capsaicin which induced neuronal death and desensitization, was recorded. The study demonstrated that ganglionated plexi sensory neurons are crucial in apnea-induced AF. Apnea led to increased GP activity, phasic bursts of vagal activity, and a tonic rise in sympathetic activity, correlating with heart rate and blood pressure changes. RTX denervation reduced nerve activity, abolished the electrophysiological response, and prevented AF inducibility during apnea [47]. Therefore, the most common mechanism of AF development in patients with OSA seems to be mixed, consisting of obstructive respiratory events triggering parasympathetic activation via the diving reflex, rapidly followed by a sympathetic spike induced by hypoxia, awakening and reduced pulmonary stretch [45]. Therefore, OSA is responsible for intensifying the density of firing premature beats originating in the pulmonary veins, which serve as the most important source of triggers for AF [48].

### 3.2. Ventricular Arrhythmias

Both OSA and central sleep apnea (CSA) may contribute to nocturnal cardiac arrhythmias. Data on the prevalence of clinically significant nocturnal atrial and ventricular arrhythmia in patients with heart failure with reduced ejection fraction (HFrEF) and OSA or CSA are scarce. In patients with HFrEF, the prevalences of nocturnal ESVEA, AF, and PVC > 10/h were higher in those with OSA or CSA than in those without OSA or CSA, and OSA severity was related to the burden of nocturnal atrial ectopy. The severity of OSA or CSA was not significantly associated with AF or > 10 PVC/h [49]. An experimental study provided intriguing insights by comparing the effects of CSA and OSA on ventricular repolarization in both humans and pigs. The study concluded that in patients with OSA, unlike those with CSA, the negative fluctuations in intrathoracic pressure and the activation of the sympathetic nervous system led to the prolongation and dispersion of ventricular repolarization time [50]. Variations in intrathoracic pressure during obstructive apneas induce alterations in ventricular repolarization that are absent in central apneas. These alterations are primarily mediated by sympathetic nervous system activation and may underlie the elevated risk of sudden cardiac death [50]. The complexity of OSA, including upper airway dysfunction, leads to cardiovascular changes such as blood pressure elevation, impaired vasoreactivity, functional ischemia and cardiac remodeling, all of which can contribute to the development of ventricular arrhythmias [51]. During OSA, there are acute surges in sympathetic nervous system activity. However, this heightened activation persists throughout the day, manifesting as increased daytime muscle sympathetic nerve activity and elevated plasma catecholamine levels in OSA patients [52]. An animal model experimental research explored the susceptibility of endocardial cardiomyocytes to intermittent hypoxia (IH), which leads to variations in ion channel expression affecting action potential duration (APD). Specifically, IH upregulated Cav1.2, TRPC1, and TRPC6 in endocardial cells, mediated by the transcription factor HIF-1. This upregulation can prolong endocardial APD, increase QTc and Tpeak-Tend intervals and lead to calcium overload, which predisposes to arrhythmogenic early afterdepolarizations and premature ventricular contractions. The sympathetic nervous system activation further affects these changes as catecholamines enhance the activity of LTCC and TRPC channels, promoting calcium overload-related ventricular arrhythmias [53].

### 3.3. Sudden Cardiac Death

Chronic exposure to intermittent hypoxia has been shown to modify ventricular repolarization, resulting in prolonged QTc and Tpeak-Tend intervals. An increased QTc interval serves as an indicator of ventricular electrical instability and is associated with an increased risk of ventricular arrhythmias and sudden cardiac death. Additionally, the prolonged Tpeak-Tend interval, believed to indicate transmural dispersion of ventricular repolarization, is considered an even more significant predictor of arrhythmia-related SCD in individuals with cardiovascular disease [53].

One review focused on the association between OSA and sudden death in non-cardiac populations aiming to assess whether OSA increased the risk of sudden death in individuals without pre-existing heart conditions. The review found evidence linking OSA to cardiac arrhythmias and increased risk of sudden death in individuals with pre-existing heart disease. However, they found limited evidence to support OSA as an independent risk factor for sudden death in those without heart disease [54].

### 3.4. Bradyarrhythmias

Sinus node disease, including bradycardia with chronotropic incompetence, sinoatrial block, and tachycardia-bradycardia syndrome, were found more frequently in the sleep apnea syndrome population compared to the general population. The increased parasympathetic tonus present in patients with sleep apnea has been incriminated as the primary mechanism of bradyarrhythmias. Also, the duration and severity of bradyarrhythmia episodes were correlated with the degree of hypoxemia during apneic events [55]. When hypoxemia is accompanied by apnea, the adaptative responses of the sympathetic and vagal systems become more pronounced, resulting in enhanced sympathetic vasoconstriction and vagal bradycardia [56]. Initially, apnea leads to an increase in vagal tone, which is subsequently replaced by an abrupt sympathetic discharge once hypoxia and hypercapnia set in. Following vagal activation, which is associated with the shortening of myocardial ERP, the sympathetic discharge is highly likely to induce arrhythmias [55]. 

A recent meta-analysis demonstrated a significant comorbid disease burden between OSA and bradycardia [57]. The association between the most frequent arrhythmias, classified by different ECG change types, and OSA is represented in Table 1. In summary, AF’s strong association with OSA is supported by a meta-analysis with an odds ratio indicating twice the risk of AF in patients with OSA [42]. 

Bradycardia is associated with OSA on a Level 1 basis, as suggested by data from a recent systematic review and meta-analysis. In contrast, the association between ventricular arrhythmias and OSA, as well as SCD and OSA, are based on non-randomized studies and animal studies suggesting levels 2 and 3 of evidence, respectively.

## 4. Screening and Clinical Evaluation

### 4.1. Screening OSA

Current studies concluded that the recommendation for screening for OSA goes beyond those with obvious symptoms, highlighting the importance of proactive screening in high-risk individuals. These high-risk categories include individuals with resistant hypertension and pulmonary hypertension, which frequently overlap with OSA. Recurrent AF that persists despite interventions such as cardioversion or ablation is another critical indicator. The rationale behind recommending screening even with only a suspicion of sleep-disordered breathing or excessive daytime sleepiness is the potential to prevent serious cardiac events. This proactive approach aims for early detection and management to mitigate long-term cardiovascular complications [1]. The European Society of Cardiology guidelines recommend that SDB be considered in the differential diagnosis of bradyarrhythmias [6]. Also, current guidelines strongly consider screening patients with tachy-brady syndrome or ventricular tachycardia and survivors of sudden cardiac death if OSA risk factors are present, as the early diagnosis and appropriate management of OSA may reduce associated cardiovascular risks [55].

An observational study by Becker et al. found that the prevalence of bradyarrhythmias correlated strongly with OSA severity and degree of nocturnal desaturations [58].

Mehawej et al., aimed to examine the associations between OSA and AF-related frailty, cognitive performance, and quality of life among older adults with AF resulting that participants at intermediate or high risk of OSA were more likely to be frail, thus identifying a group of high-risk patients who would benefit from early screening for OSA [59].

Screening for OSA involves utilizing medical history, questionnaires (like the Berlin Questionnaire, STOP-BANG, and Epworth Sleepiness Scale), and sleep apnea screening devices. While these tools possess varying degrees of sensitivity and specificity, they are crucial in identifying individuals at risk. Importantly, screening instruments may underperform in certain populations, including women and patients with pre-existing cardiovascular conditions.

A detailed medical history (including the patient’s partner as he or she can provide important information about what occurs during the night) and clinical examination are important in the evaluation of patients suspected of having OSA [60]. The Mallampati classification (examination of the oropharyngeal inlet) is also used to assess whether the enlargement of the tonsils, uvulae, and tongue affect airway volume, an important aspect in terms of therapeutic management in the presence of sleep apnea syndrome [61].

Current screening methods, while useful for initial assessment, fail to provide definitive diagnoses. The STOP-BANG and Berlin questionnaires, while convenient and widely used, are known to have limitations, primarily low specificity. A low specificity means that a positive test result does not definitively confirm OSA, generating false positives. Many individuals testing positive on these questionnaires require further testing to rule out OSA. This necessitates additional diagnostic procedures, leading to potential delays in treatment and increasing healthcare costs [1].

The STOP-BANG questionnaire includes questions on snoring, tiredness, observed apneas, blood pressure, BMI, age, neck circumference, and gender. It is one of the most sensitive questionnaires available for use in the clinical setting. Every parameter is given one point, and a score of more than 3 indicates a high risk of OSA [61].

The Berlin questionnaire includes snoring history, degree of fatigue, documented apnea, and history of hypertension and obesity. Each question falls into one of three categories and each category has its point system. The first category asks about snoring and observed apneas; the second category examines the degree of fatigue; and the third category asks whether the patient has hypertension and includes the patient’s BMI. A patient is classified as having a low risk of OSA if they have one or no categories with a positive score and a high risk if two or more categories are positive. The Epworth Sleepiness Scale focuses on daytime sleepiness, a common OSA symptom. This eight-item questionnaire administered during a clinical encounter and can prove useful in establishing whether significant OSA symptoms are present. However, many individuals with OSA do not experience excessive daytime sleepiness, so the scale misses a significant number of cases (low sensitivity) [62].

A validated OSA screening instrument for patients with paroxysmal AF holds promise. Specifically, incorporating NABS (Neck Circumference, Age, Body Mass Index, and Snoring) had improved discriminative ability compared with commonly used instruments such as STOP-BANG [6].

All these screening tools may be less effective in certain populations, such as women, who may present with fatigue and insomnia rather than sleepiness, and in individuals with pre-existing cardiovascular conditions. A prospective study that compared the performance of seven screening tools (Epworth Sleepiness Scale, Berlin Questionnaire, Clinical Sleep Apnea Score, NoSAS, OSA50, STOP-Bang, and MOODS) with polysomnography in detecting clinically relevant OSA in patients with AF showed that none of the selected screening tools demonstrated sufficient performance as a good discriminative screening tool for clinically relevant OSA in patients with AF [63].

**Figure 2:**
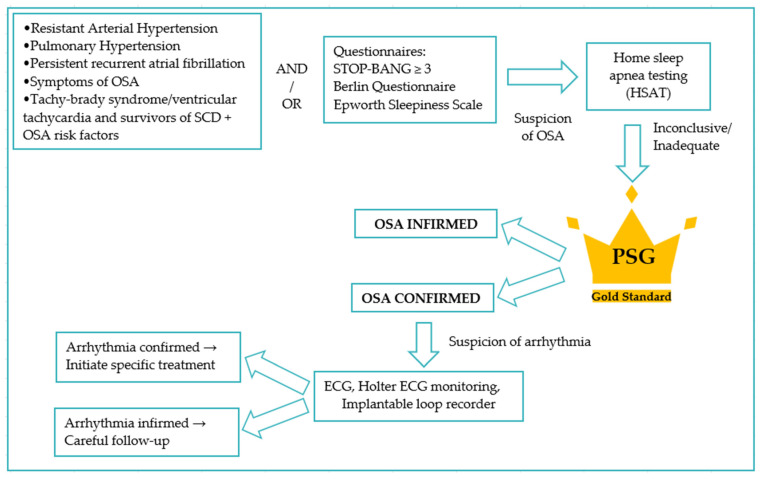
Screening and investigation algorithm for patients at risk or suspected of obstructive sleep apnea in relation to arrhythmia.

The diagnostic gold standard remains in-laboratory polysomnography (PSG) but its cost, invasiveness, and limited accessibility are significant barriers to widespread use (Figure 2).

However, home sleep apnea testing (HSAT) offers a more accessible alternative, particularly with level 3 HSAT demonstrating excellent diagnostic accuracy. Level 3 HSAT typically measures oxygen saturation (SpO2), respiratory effort, airflow, and heart rate, enabling a more thorough assessment than some simpler home testing methods. The advantages are not only increased diagnostic accuracy but also convenience, affordability, and improved patient compliance, contributing to earlier diagnosis and treatment initiation. The first study to validate a level 3 HSAT (also known as polygraphy) in an AF population, indicating its superiority to other OSA screening tools, was published in 2021 by Mohammadieh AM et al. [40]. Overall, a level 3 HSAT showed the highest diagnostic accuracy at all levels of OSA severity: mild, moderate, and severe [40]. However, it may not capture all aspects of sleep architecture and may be less accurate in detecting milder forms of OSA or comorbid sleep disorders [15]. Therefore, if an HSAT is inconclusive, inadequate, or negative, PSG should be performed. Patients with comorbidities such as cardiovascular disease, respiratory muscle weakness secondary to neuromuscular disorders, history of strokes or other ischemic disease, and chronic opioid use should undergo PSG rather than HSAT [61]. PSG is considered the gold standard for the diagnosis of sleep disorders owing to its multi-channel data acquisition, which includes brainwave activity and cardiac telemetry to allow for sleep staging, arousal assessment, and assessment of heart rate variability [55].

The AHI is the most common metric used to quantify OSA severity. While AHI is widely used and relatively easy to calculate, it has limitations. AHI, when considered in isolation, might not fully reflect the actual severity of OSA, as it is common to see patients with low AHI and high levels of symptoms, and it is also quite common to see patients with AHI >30/h and very low levels of symptoms. AHI does not take into account important aspects of respiratory events such as the magnitude of the associated desaturation or the presence of awakenings [64].

Some studies suggest that measures of oxygen desaturation might be better indicators of OSA’s impact on cardiovascular health and that these desaturation indices (like T90 and T88) are more strongly associated with adverse cardiac events [55]. A study in Cyprus was conducted to examine possible associations between sleep respiratory indices and cardiovascular disease in OSA. The study concluded that AHI was the defining factor of sleep correlating with heart failure. In addition, the risk of heart failure was associated with AHI ≥ 15 and was highly statistically significant [65].

A large clinic-based cohort study by Gami et al., examined the predictive value of the lowest nocturnal oxygen saturation on the risk of sudden cardiac death (SCD) [66]. Unlike some previous studies, this study found that the lowest nocturnal oxygen saturation was an independent predictor of SCD; every 10% decrease in the lowest O2 saturation correlated to a 14% increase in SCD risk. Traditional metrics like AHI were not predictive of SCD in this study. It is important to note that the authors acknowledge that this modest association might be influenced by underlying cardiopulmonary conditions or body habitus. This study was one of the first to directly link OSA to increased SCD risk [67].

Another sleep-disordered breathing, central sleep apnea, occurs because the brain does not send proper signals to the muscles that control breathing. Central sleep apnea is not frequently diagnosed in the active-duty population. Still, it is increasing in the older population, especially in those with comorbidities like heart failure, stroke, neuromuscular disorders, and opioid use. Also, it is associated with increased admissions related to comorbid cardiovascular disorders and an increased risk of death [68].

### 4.2. Screening Arrhythmia in SDB

AF is the most frequent chronic arrhythmia globally, associated with substantial morbidity and mortality. Researchers emphasize the increasing prevalence of AF due to an aging population and the increasing incidence of predisposing risk factors. These risk factors are listed as hypertension, coronary artery disease, heart failure (including heart failure with preserved ejection fraction, chronic kidney disease, obesity, OSA, and even psychological factors such as anxiety. The study by Mariani MV et al., predicts a substantial increase in the number of AF cases in the US and Europe by 2050 and 2060, respectively, highlighting the growing healthcare burden associated with the condition and its frequent presentation to emergency departments [69]. The study does not address any specific treatments or prevention strategies for AF but it highlights the importance of tackling its risk factors.

Multiple other studies demonstrate a significantly higher prevalence of AF and other arrhythmias in OSA patients compared to the general population. The severity of OSA correlates with the increased risk and frequency of arrhythmias. Arrhythmias can exacerbate symptoms of breathlessness and fatigue in patients with OSA and can also lead to stroke, sudden cardiac death (SCD), and heart failure [70]. A study conducted by Acharya R et al., showed that mortality was significantly higher across all categories of arrhythmias and conduction disorders when associated with OSA in comparison to non-OSA patients [71]. One other study showed that 700,000 people in the United States may have undiagnosed AF, with an estimated cost burden of 3.2 billion dollars. It is well known that asymptomatic AF is associated with a similar risk of all-cause death, cardiovascular death, and thromboembolism compared with symptomatic AF [72].

May et al. revealed how different indices of SDB predict the occurrence of new AF in a cohort of 843 elderly adults who were initially AF-free. During a mean follow-up of 6.5 years, 12% of participants developed AF. The study also identified central sleep apnea and Cheyne-Stokes breathing as significant predictors of AF [73].

Also, a longitudinal cohort study involving 10,701 adults investigated the connection between OSA and increased risks of SCD. The study found that people with an AHI > 20 and nocturnal oxygen saturations below 78% had a significantly increased risk of SCD [73].

The “Association of Short Sleep Duration and Atrial Fibrillation” study analyzed the association between sleep duration and AF in a large cohort of 31,079 patients who underwent diagnostic polysomnography. This study showed that for every one-hour reduction in total sleep duration, there was a 17% increase in the risk of prevalent AF and a 9% increase in the risk of incident AF; the study concluded that patients who slept less than three hours were 2.10 times more likely to have prevalent AF than those who slept more than six hours [73].

In a meta-analysis of sixteen observational studies, the odds ratios for new onset AF for no obvious reason, new onset AF after surgical operations, such as coronary artery bypass grafting, and AF after ablation treatment were 1.71, 2.65, and 2.93, respectively. Linear dose–response meta-analysis results revealed that the risk of AF increased with increasing AHI value [74]. Hypertensive patients with AHI > 15 are at an increased risk for atrial arrhythmias and left atrial dilation, with HRV significantly correlating with OSA severity [75].

Significant night-to-night variability in OSA severity has been shown in AF patients, which has implications for diagnostic testing [76]. An investigation of night-to-night variability in OSA severity found that nights with the highest severity had an increased risk of having AF during the same day [77].

Non-linear analysis, incorporating machine learning (ML) and artificial intelligence, has the capacity to identify patterns and make predictions regarding OSA that traditional analytical methods may overlook. Recent research on the integration of ML models in the field of sleep-related breathing disorders indicates substantial promise. The study demonstrates that advancements in wearable sensor technology, when combined with ML techniques, provide clinicians with the capability to predict, diagnose, and classify OSA with enhanced accuracy and efficiency. This innovative approach serves as a valuable tool in the ongoing efforts to improve the management and treatment of OSA, ultimately contributing to better patient outcomes and healthcare practices [78].

In the Tokyo Sleep Heart Study, a large Japanese sleep cohort designed to determine clinical predictors of AF in patients with OSA, the CONUT score was found to be significantly associated with the presence of AF in patients with OSA. CONUT scores were calculated from total peripheral lymphocyte count, serum albumin level, and total cholesterol level. Moreover, Furui et al., observed that the recurrence rate of AF after catheter ablation was higher in undernourished patients compared to those with normal nutrition [79].

A study conducted by Stafford PL et al., on positional OSA found that this condition is common, affecting more than half of AF patients, implying that positional therapy should be considered in patients with AF, mild and positional OSA [80].

Some studies found variations in the association between OSA, arrhythmias, and other cardiovascular outcomes across different sexes and ethnic groups. The Multi-Ethnic Study of Atherosclerosis (MESA) provided insight into these relationships by analyzing over 2200 individuals from diverse racial and ethnic backgrounds and showed that African Americans had significantly higher odds of sleep apnea syndrome. Also, women, for example, showed a different risk profile than men. The studies also identify gaps in current screening methods and suggest avenues for improving diagnosis and treatment to improve cardiovascular outcomes in OSA patients. The data also highlight the importance of considering sex and ethnicity when investigating the OSA-arrhythmia relationship [73].

Arrhythmias are detected by ECG or ambulatory ECG monitoring (e.g., Holter monitoring). However, these diagnostic tools can often misdiagnose significant paroxysmal arrhythmias. 24 h Holter recording provides information related to sympathetic and parasympathetic activity using spectral analysis of heart rate variability (HRV). Variations in HRV patterns (ultra-low, very-low, low, and high-frequency bands) indicate a compromised ability of the autonomic nervous system to effectively regulate the heart, leading to a heightened risk of arrhythmias. The analysis of Holter data also provides information related to heart rate turbulence. Analysis and comparison of HRV before initiation of CPAP treatment and at a time interval after treatment will demonstrate any significant changes in cardiac autonomic control during this period [70].

Implantable loop recorders are powerful tools for long-term arrhythmia detection. They are small devices used to detect arrhythmias. Once implanted, they continuously monitor heart rhythm and record abnormalities for 3 years or more. The data stored on the device is transmitted via Bluetooth to a base station connected to a mobile phone which then sends the information to the database. Unlike Holter monitoring, they provide a complete extended temporal profile [70]. One study using implantable loop recorders found AF in 20% of OSA patients, a rate far exceeding that of the general population (up to five times higher) [70].

### 4.3. Clinical Implications

The first study suggesting an association between OSA and SCD was carried out by Gami et al., in 2005 [81]. It found a significantly higher incidence of SCD during nocturnal hours (midnight to 6 a.m.) among OSA patients. This nocturnal increase in SCD risk is noteworthy because cardiovascular events typically show a circadian pattern, with lower occurrence during sleep. The discrepancy highlights the unique and potentially dangerous interaction between OSA and cardiac risk [67]. In addition to this, a meta-analysis by Wang et al., significantly reinforces the findings of Gami’s research by indicating a dose–response relationship: increased OSA severity translates to higher risks of cardiovascular disease, stroke, and all-cause mortality [82]. These findings underscore OSA’s critical role as a modifiable risk factor for significant cardiovascular complications and death.

There are some electrocardiographic (ECG) markers indicative of increased risk:

Increased QT dispersion (the difference between the longest and shortest QT intervals on an electrocardiogram), particularly QTcd exceeding 60 ms, is a strong independent risk factor for cardiac mortality. Studies show a tendency for QTcd to increase during sleep in OSA patients without hypertension, although this is not universally observed. However, a contradictory study (Barta et al.) found no such increase during sleep, suggesting a potential influence of factors not accounted for in the studies, like AHI levels or other comorbidities. The variability highlights the complex interplay between OSA, sleep stage, and QT dispersion [60].

Prolongation of the TpTe interval (the time between the T-wave peak and end on an ECG) is associated with an increased risk of ventricular tachycardia and SCD. Studies show a tendency towards increased TpTe in OSA patients, possibly linked to negative intrathoracic pressure changes during apneas [60].

The presence of fragmented QRS, reflecting disordered ventricular depolarization in OSA patients, independent of obesity, suggests electrical myocardial remodeling, a significant finding indicating structural cardiac changes associated with OSA [60].

A cross-sectional study aimed at identifying the main risk factors for OSA concluded that OSA, while univariate analyses found associations with hypertension, diabetes, dyslipidemia, age, and BMI, only age and BMI remained significant in multivariate logistic analysis [83].

Gut microbiota, inflammation and oxidative stress may underlie the high comorbidity of OSA with metabolic disorders and other comorbidities such as obesity, cardiovascular problems, insomnia [84].

The gut-derived metabolite trimethylamine N-oxide (TMAO) was identified as a key player in increasing cardiac sympathetic nervous system activity and promoting arrhythmias [76].

The analysis of a large dataset identified two microbial taxa, Eubacterium ramulus and Holdemania, as causally associated with AF. Further analysis suggested that coronary artery disease mediates the effect of Eubacterium ramulus on AF, while BMI mediates the effect of Holdemania. The study provided genetic evidence supporting a causal link between specific gut microbiota and AF risk, highlighting potential therapeutic avenues through targeting the gut microbiome [85].

A two-sample Mendelian randomization study investigated the causal relationship between gut microbiota and both arrhythmias and conduction blocks. Gut microbial taxa was associated with an increased or decreased risk of specific arrhythmias. For example, Ruminococcaceae UCG004 showed a negative correlation with AF, while Holdemania was associated with a reduced risk of paroxysmal tachycardia. For atrioventricular block the order Bifidobacterbacteriales, family Bifidobacteriaceae and genus Alistipes showed a negative correlation, while genus Candidatus Soleaferrea showed a positive correlation. In regard to left bundle branch block, the family Peptococcaceae appeared to reduce the risk, while the genus Flavonifractor was linked to an increased risk. Finally, no causal gut microbiome was identified in the context of right bundle branch block [86].

A study by Ramphul et al., confirmed that patients with OSA had a lower risk of dying when hospitalized with acute myocardial infarction or acute ischemic stroke in the United States. Similar results were also previously described by Mohananey et al. for outcomes of STEMI among OSA patients and by Lapow et al., for patients admitted for acute ischemic stroke. The lower mortality rate can be attributed to a possible higher quality of care and more aggressive treatment protocols for patients who have already been previously diagnosed with OSA [87]. According to a recent meta-analysis, OSA was also associated with a significant overall increase in the risk of aortic dissection by 60%, possibly due to mechanisms of sympathetic vasoreactivity and inflammation induced by intermittent hypoxia. Moreover, it has also been reported that patients with moderate or severe OSA have an increased risk of aortic dissection by up to 44%, implying that the severity of OSA has a positive correlation with the risk of aortic dissection. OSA appears to act as a risk factor for the development of both heart failure with reduced ejection fraction (HFrEF) and heart failure with preserved ejection fraction (HFpEF) [88].

Shiao et al. explored the predisposition of patients with peptic ulcer bleeding to have sleep apnea and stated that sleep apnea was likely an independent risk factor for peptic ulcer bleeding. The underlying mechanisms for this may be that sleep apnea participates in the occurrence and development of peptic ulcers and peptic ulcer bleeding through mechanisms like intermittent hypoxia, systemic inflammation, oxidative stress, and sympathetic activation [89].

A review performed by Chaitanya et al. highlights OSA as a risk factor for developing glaucoma. Another important point to note is that CPAP therapy can trigger glaucoma damage by raising the intraocular pressure, which would indicate glaucoma screening in patients on CPAP. A study investigating the association between OSA and middle ear acoustic cochlear function demonstrated that severe OSA is associated with cochlear function impairment [61].

One retrospective study aimed to determine whether some polysomnographic parameters (e.g., apnea-hypopnea duration, sleep structure, nocturnal hypoxemia) were specifically correlated with cardiometabolic comorbidities in OSA. It was concluded that patients with more severe OSA, longer sleep apnea and hypopnea, and marked intermittent/global nocturnal hypoxemia were more likely to develop cardiometabolic comorbidities [90]. The same conclusions were obtained by Kainulainen S et al., emphasizing the need for further studies to identify the added value of comorbidities in determining patient prognosis [91].

## 5. Prevention and Treatment Alternatives of Arrhythmia Associated with OSA

To effectively manage arrhythmia in patients diagnosed with OSA, it is crucial to ensure optimal respiratory flow during sleep, thereby preventing episodes of hypoxia and hypercapnia. The main therapeutic approach is the implementation of CPAP therapy.

CPAP therapy is acknowledged as the gold standard treatment but is often poorly tolerated due to discomfort, claustrophobia, and difficulty adapting to its use. Many patients experience mask leaks, dry mouth, and nasal congestion. Conventional PAP therapy, CPAP, and bilevel PAP therapy are considered standard forms of PAP. Findings from studies show that a low ArTH (a measure of upper airway resistance) is associated with lower PAP adherence, particularly in specific populations (veterans, post-stroke patients, and those with coronary artery disease). The same study suggests finding strategies for improving PAP adherence, including motivational interviewing and addressing comorbidities like insomnia [92]. A meta-analysis of seven randomized controlled trials, which included 4268 patients, showed a significant reduction in relative risk or major adverse cardiovascular events and stroke, which correlated with increased CPAP usage time (adherence time >4 h) [93].

Some studies demonstrate a significant reduction in AF recurrence with CPAP, while others show mixed results. The consistency of positive findings across numerous meta-analyses; however, points toward a noteworthy benefit of CPAP in reducing the burden of AF recurrence, regardless of whether the patient received additional interventions like pulmonary vein isolation (PVI). This suggests that CPAP may target underlying proarrhythmic mechanisms linked to OSA [58]. Abe et al., conducted a study of 1394 Japanese patients and reported a significant reduction in the incidence of AF, PVCs, sinus bradycardia, and sinus pauses with CPAP therapy [93].

A meta-analysis investigated the effects of CPAP therapy on major cardiovascular events in patients with moderate to severe OSA and coronary artery disease. The study analyzed data from 11 trials involving 5410 patients, finding that CPAP therapy was associated with a modest but statistically significant reduction in the risk of major cardiovascular events (23% reduction) and all-cause cardiovascular death (23% reduction). CPAP therapy also demonstrated a positive effect on systolic and diastolic blood pressure. The meta-analysis found no statistically significant effects of CPAP therapy on arrhythmia, acute coronary syndrome or rehospitalization for heart failure [94].

CPAP therapy often does not fully address all aspects of OSA, and additional therapies are often required.

### 5.1. Pharmacotherapy

Anti-depressants like Fluoxetine are SSRIs that have been studied as a method of treating OSA considering that sleep-dependent serotonin delivery is responsible for stimulating upper airway dilator motor neurons. Hanzel et al. found that mean AHI decreased from 57 to 34 events per hour in patients treated with Fluoxetine, revealing Fluoxetine as a potential pharmacological solution for increasing the patency of upper airway muscles during sleep [95].

Pre-clinical and smaller human studies have shown that drugs with both noradrenergic (affecting norepinephrine, a neurotransmitter that increases arousal and muscle tone) and antimuscarinic (blocking muscarinic acetylcholine receptors, which can relax muscles in the upper airway) properties can improve genioglossus muscle activity during sleep (the genioglossus muscle is crucial for maintaining airway patency). The MARIPOSA randomized controlled trial, which investigated the efficacy and safety of AD109 (a combination of atomoxetine and aroxybutynin) in treating OSA, concluded that AD109 produced a “clinically meaningful improvement” in OSA parameters. This is a significant finding because effective and well-tolerated pharmacological treatments for OSA have been largely unavailable. The implication is that AD109, or similar drugs, could provide a less invasive alternative or adjunct therapy to CPAP. Further development and more extensive clinical trials are warranted to confirm these findings and assess their long-term effects and safety profile [96]. The SURMOUNT-OSA trials assessed the effects of tirzepatide, a GIP/GLP-1 receptor co-agonist (a drug that mimics the action of gut hormones to regulate blood glucose and appetite), on OSA and obesity. The results showed that the change in the apnea-hypopnea index is clinically relevant, but its impact on cardiovascular mortality remained unclear [96].

### 5.2. Mandibular Advancement Devices

Several studies discuss various treatments for OSA, focusing on oral appliance therapy, specifically mandibular advancement devices, and comparing their effectiveness to CPAP. A recurring theme is the importance of patient selection to maximize treatment success and minimize side effects.

Oral advancement devices aim to realign craniofacial or oral structures to enlarge the pharyngeal airway space and reduce airway collapse. Some appliances work by suspending the tongue in an anterior position in the mouth. Lazard et al., showed that a tongue-holding device can reduce the mean AHI from 38 to 14. The most common and well-studied oral advancement devices are mandibular advancement devices. Their purpose is to advance the mandible relative to the maxilla to enlarge and stabilize the upper airway. In addition, mandibular advancement devices resist the downward rotation of the mandible and associated mandibular retrusion during sleep, which compromises upper airway flow [95].

Also, some studies highlight the predictors of successful oral appliance therapy. Factors such as mild upper airway collapsibility, low genioglossus muscle activity, high arterial tonicity, and moderate upper airway collapsibility are associated with better outcomes. In contrast, factors such as age, BMI, gender, and AHI were not significant predictors of the device’s success [92]. The CRESCENT trial, a large-scale study, compared mandibular advancement devices and CPAP for blood pressure reduction in hypertensive OSA patients. It found mandibular advancement devices to be non-inferior to CPAP in reducing 24 h mean arterial blood pressure, with a trend towards the superiority of oral devices, particularly during sleep. This study included a diverse group of patients with hypertension and high cardiovascular risk, many of whom had severe OSA [97,98].

Despite the variability in adherence, mandibular advancement devices appeared to be safe and effective. However, potential downsides of oral appliances were also explored. Studies note possible side effects including temporomandibular joint issues, dental changes, and the need for regular follow-up. Other studies show that while CPAP demonstrates limited long-term dental and skeletal effects, mandibular advancement devices can lead to more significant changes. Therefore, careful patient selection and monitoring are crucial [95].

Research consistently emphasizes the need for more extensive, longer-term studies to better assess the long-term effects of different therapies for OSA and define patient selection criteria.

Hypoglossal nerve stimulation (HNS) is a surgical procedure where a stimulator is connected to the hypoglossal nerve, triggering genioglossus muscle contraction to prevent upper airway collapse during sleep [99]. The FDA approved HNS in 2014, and studies show significant improvements in quality of life, AHI, and oxygen desaturation index following the procedure. Long-term studies show sustained benefits for up to five years, with high patient adherence [100].

Hypoglossal nerve stimulation involves implanting a pulse generator in the upper chest with leads connected to the hypoglossal nerve and a respiration sensor. The generator delivers electrical pulses to stimulate the tongue and widen the pharyngeal wall. Post-operatively, the device is activated at night, and adjustments to electrode configurations can be made to optimize results. Although HNS offers an effective treatment option for OSA, it carries potential side effects, including incision discomfort, temporary tongue weakness, headaches, and infection [101]. Ongoing research, such as the TESLA trial, aims to explore the potential of transcutaneous electrical stimulation as a treatment modality for a wider range of OSA patients [95].

The procedure’s success relies on identifying the appropriate patient profile, which includes using tests like drug-induced sleep endoscopy (DISE) to assess the nature and location of airway collapse. The presence of complete concentric collapse is an exclusion criterion for using hypoglossal nerve stimulation. Other considerations include evaluating four key traits associated with OSA: upper airway collapsibility, ventilatory control instability, muscle responsiveness, and arousal threshold. While DISE is valuable, it involves sedation, and research is exploring non-invasive methods using baseline polysomnography to predict the site of collapse [102].

### 5.3. Surgical Treatment

Palatal surgery is a surgical treatment option for OSA patients that has known different techniques over the last couple of years.

Upper airway surgery aims to improve the anatomy of the upper airway to reduce or prevent its collapse during sleep. Uvulopalatopharyngoplasty (UPPP), a common procedure involving the removal of the uvula and parts of the soft palate, has shown some success in reducing AHI. Still, it carries risks such as bleeding, swallowing difficulties, and voice changes. Alternative palatal surgical techniques, like barbed reposition pharyngoplasty and expansion sphincter pharyngoplasty, aim to address these limitations. Non-managed OSA is a risk factor for cardiovascular diseases, including AF. While CPAP effectively treats OSA, compliance can be difficult. The study aimed to investigate whether UPPP reduces the risk of cardiovascular disease in patients with OSA and concluded that OSA raises the risk of CHF and AF, while UPPP can substantially reduce these risks in OSA patients [103].

Studies suggest that barbed reposition pharyngoplasty and expansion sphincter pharyngoplasty are often as effective as UPPP, but with fewer complications [95]. Other surgical options include midline glossectomy (partial tongue removal), which can be combined with other procedures, and tongue reduction surgery using techniques like radiofrequency ablation or transoral robotic surgery (TORS). TORS has been shown to offer high success rates with fewer complications compared to traditional methods [100].

Surgery should only be considered after less invasive treatments, such as CPAP and oral appliances, have proven ineffective. The success of surgical intervention often depends on patient selection criteria; for example, body mass index, the presence of complete concentric collapse (observed during sleep endoscopy), and the level of activity of the genioglossus muscle are significant predictors of treatment success. In general, surgical treatment is tailored to the specific anatomical problems of the individual and the severity of OSA. While surgical procedures can be very effective, they involve significant risks, and patient selection is crucial to maximize the benefits and minimize complications.

Positional therapy aims to prevent patients from sleeping on their back (supine position), as this posture exacerbates OSA. Traditional methods, such as placing a tennis ball under the back, have poor long-term adherence [95]. Newer approaches, such as vibrating devices that gently push patients out of the supine position, have better short-term adherence but lack long-term data on efficacy and potential side effects [104]. Although positional therapy may be a beneficial adjunct for some patients, it is significantly less effective than CPAP in reducing AHI and is not suitable for all patients. Studies show that positional OSA, in which AHI is significantly higher in the supine position, is reported to affect almost half of patients with OSA [100].

Beyond positional therapy, several alternative and complementary treatments are discussed. A rehabilitation program combining exercise, dietary guidance, and counseling alongside CPAP significantly improved quality of life, exercise capacity, and sleepiness in OSA patients [105]. Bariatric surgery also showed long-term benefits in reducing OSA severity, particularly five years post-surgery, with significant improvements in quality of life and a reduction in cardiovascular risk [106]. Current guidelines recommend controlling risk factors such as obesity and OSA in all patients with AF. The LEGACY study showed that weight loss alone can lower the burden of AF and help maintain sinus rhythm. An important aspect of this study was that a reduction in body weight of more than 10% was associated with a 6-fold greater likelihood of freedom from arrhythmias in patients with OSA [62].

Treatment selection should consider individual patient characteristics, preferences, and the severity of their condition. While positional therapy can play a supportive role, it is crucial to carefully consider CPAP, surgical interventions, oral appliances, and other therapies to optimize long-term treatment outcomes. More research is needed on the long-term efficacy and side effects of many of the discussed treatments [104].

Antiarrhythmic drug therapy. There is a lack of evidence supporting the efficacy of antiarrhythmic drug therapy or the superiority of any specific antiarrhythmic drug class in patients with OSA. However, several small-scale studies indicated that patients with severe OSA have a reduced responsiveness to antiarrhythmic drugs compared to patients without OSA [107].

### 5.4. Catheter Ablation or Cryoablation Therapy

There seems to be a bidirectional relationship between AF and OSA, indicating that screening for AF in patients with sleep disorder breathing may be crucial, particularly for identifying asymptomatic AF and nocturnal episodes. Early detection through screening can inform and establish the need for anticoagulation and antiarrhythmic treatment strategies. An array of observational studies and meta-analyses have indicated that OSA is associated with an increased risk of recurrent AF following cardioversion and catheter ablation procedures. This elevated risk appears to be diminished by the effective use of CPAP therapy. Nonetheless, the question of whether OSA treatment can significantly reduce the overall burden of AF remains unresolved due to a lack of compelling evidence from randomized controlled trials. To date, only two small randomized controlled trials have examined the isolated impact of CPAP therapy on AF burden after cardioversion and ablation, with neither study showing a substantial reduction in AF recurrence [41,108,109]. The severity of OSA has been found to correlate with poorer outcomes following ablation procedures. This suggests that patients with more severe OSA may experience less favorable results and a higher likelihood of recurrent AF after undergoing ablation [110,111]. Following recent recommendations by the American Heart Association, risk factor modification should become an essential complementary cornerstone of AF management, together with anticoagulation, rhythm, and rate control. This approach incorporates screening and treatment of OSA, especially before recommending pulmonary veins isolation [112]. In relation to ventricular arrhythmia, a recent study showed a significant association between the genesis and recurrence of right ventricular outflow tract post successful radiofrequency ablation and the presence of OSA. Moreover, the study indicated that patients with OSA are more susceptible to recurrent arrhythmias following radiofrequency catheter ablation if OSA is not effectively managed [113].

### 5.5. Implantable Cardioverter-Defibrillator (ICD) and Other Device Therapy

The AIRLESS study demonstrates that the transthoracic impedance sensor, utilizing the ApneaScan algorithm, integrated into ICDs for sleep apnea detection by evaluating the transthoracic impedance, is a reliable tool for identifying patients suffering from severe sleep disorders. This novel technology holds potential for enhancing the clinical management of this patient population, leading to improved diagnostic accuracy and treatment outcomes [114]. A study conducted by Mazza et al. used the ICD-computed Respiratory Disturbance Index (RDI) to identify patients with severe sleep apnea. This study revealed a significant association between higher RDI values, indicative of more pronounced sleep-disorder breathing periods and the occurrence of appropriate shocks for life-threatening arrhythmias [115]. OSA is also reliably detected by pacemakers using RDI measurements [116].

There appears to be an increasing awareness regarding the reevaluation of the necessity for permanent pacing in patients with bradycardia and high-degree atrio-ventricular block who have associated OSA, particularly following CPAP therapy. This growing recognition highlights the importance of reassessing the need for long-term pacing interventions in light of the potential improvements in cardiac function and overall health that may result from effective OSA management through CPAP therapy [117].

OSA increases the risk of congestive heart failure (CHF) and AF. Within this population, UPPP can significantly reduce the risk of cardiac complications in patients with OSA. Non-managed OSA is a risk factor for cardiovascular diseases, including AF. Although CPAP therapy is effective in treating OSA, patient compliance can be challenging. The study aimed to investigate whether UPPP reduces the risk of cardiovascular events in patients with OSA. It concluded that OSA raises the risk of CHF and AF, while UPPP can substantially mitigate these risks in OSA patients.

According to the previously mentioned research, Table 2 illustrates the current levels of evidence concerning the relationship between different treatment modalities for OSA and arrhythmic events.

This narrative review has several limitations, including the variability and geographical disparities in the data on OSA. Studies examining regional, gender-based, and daily or seasonal variability highlight the importance of contextual factors in shaping the clinical profile and management for OSA patients [118,119,120,121]. These variations necessitate cautious interpretation when generalizing findings across diverse populations and emphasize the need for tailored approaches to effectively address the unique challenges for different populations. Future research should aim to address these discrepancies by incorporating more geographically representative data and fostering international collaborations. Furthermore, the lack of standardized approaches to managing OSA, particularly its cardiac complications, underscores the need to develop globally accepted guidelines to ensure equitable and effective patient care.

## 6. Conclusions

This review aimed to summarize the substrate of cardiac arrhythmias as revealed over time and provide an accurate overview of the latest management options for both sleep apnea and cardiac arrhythmias. Our exploration indicates that the most frequently implicated mechanisms generated by OSA and linked to arrhythmia genesis include hypoxia, hypercapnia, functional ischemia, nocturnal intrathoracic pressure variations, autonomic nervous system alterations with abnormal sympathetic activation, chemo- and baroreflex alterations, inflammation, and cardiac remodeling. The most frequently encountered arrhythmias in patients with OSA include AF, ventricular arrhythmias potentially associated with sudden cardiac death, and bradyarrhythmias requiring permanent pacing therapy. Screening for OSA and arrhythmias in patients with OSA provides essential information regarding the necessity for further interventions such as CPAP therapy, anticoagulation, antiarrhythmic drug therapy, catheter ablation of certain arrhythmias, or device therapy. Other extracardiac OSA-related pathologies, such as peptic ulcer disease, aortic dissection, and cardiometabolic disorders, can potentiate cardiovascular damage. Emerging therapies for OSA treatment could significantly impact arrhythmia genesis in patients with sleep-disordered breathing, but further research is needed to validate these findings and develop comprehensive treatment protocols.

### 6.1. Existing Research Gaps

Positive airway pressure remains the mainstay of treatment but unfortunately is associated with poor adherence patterns. Even though other options like oral appliances and stimulation devices are available in the market, they lack evidence demonstrating reductions in cardiovascular morbidity and mortality. Further study is required to fully elucidate the favorable effects of OSA treatment modalities on cardiovascular diseases [122].

What has yet to be clearly established is whether OSA treatment—and CPAP treatment in particular—actually improves rhythm-related outcomes in patients with OSA. Some RCTs looking at the effect of CPAP treatment on cardiac outcomes have been admittedly disappointing, possibly because these trials enrolled patients with few or no OSA symptoms for ethical reasons, and many of these trials studied patients who were much less obese than the average “real-world” OSA patient [109].

### 6.2. Potential for Future Studies

Future RCTs, appropriately powered, should look to include sleepy patients with higher BMIs to see if a lack of treatment effects persists [55].

The potential role of a multi-faceted intervention for OSA, such as combining CPAP with structured weight loss, lifestyle and nutritional counseling, and exercise, deserves more investigation, as there are data that suggest that these approaches may benefit patients with heart rhythm disorders more than CPAP therapy alone [84].

## Figures and Tables

**Table 1:** Levels of Evidence for the Association between Common ECG Changes and OSA.

ECG Change Type	Association with OSA	Level of Evidence
Atrial Fibrillation	Strongly associated	High (level 1)
Bradycardia	Associated	High (level 1)
Premature Ventricular Beats/ Ventricular Tachycardia	Associated	Moderate (level 2)
Malignant Ventricular Arrhythmias responsible for SCD	Associated	Moderate (level 3)

**Table 2:** Evidence Levels on the Effect of Different OSA Treatments on Arrhythmic and Other Cardiovascular Events. CPAP—continuous positive airway pressure; OSA—obstructive sleep apnea; AHI—apnea-hypopnea index; ICD—implantable cardioverter-defibrillator.

Treatment Modality	Level of Evidence	References
Continuous Positive Airway Pressure (CPAP)	High	Meta-analysis of 7 randomized controlled trials with 4268 patients showed a significant reduction in cardiovascular events and stroke (adherence time > 4 h) Meta-analysis of 11 trials with 5410 patients showing significant reduction in major cardiovascular events and all-cause cardiovascular death
Pharmacotherapy	Moderate	MARIPOSA randomized controlled trial, SURMOUNT-OSA trials
Mandibular Advancement Device	Moderate	CRESCENT trial comparing mandibular advancement devices and CPAP for blood pressure reduction
Hypoglossal Nerve Stimulation	Moderate	Research showing significant improvements in quality of life, AHI, oxygen desaturation index, and sustained benefits for up to 5 years
Surgical Treatment (Uvulopalatopharyngoplasty)	Moderate	Studies showing success in reducing AHI but with risks such as bleeding, swallowing difficulties and voice changes
Bariatric Surgery	Moderate	Long-term benefits in reducing OSA severity, quality of life improvement and reduction in cardiovascular risk 5 years post-surgery
Positional Therapy	Low	Traditional methods less effective than CPAP in reducing AHI, adherence issues
Antiarrhythmic Drug Therapy	Low	Lack of evidence supporting efficacy, reduced responsiveness in severe OSA patients
Catheter Ablation or Cryoablation Therapy	Moderate	Observational studies and meta-analyses indicate increased risk of recurrent AF following ablation procedures, poorer outcomes with severe OSA
Implantable Cardioverter-Defibrillator (ICD) and Other Device Therapy	Moderate	AIRLESS study demonstrates reliable detection of OSA through integrated transthoracic impedance sensor

## Abbreviations

The following abbreviations are used in this manuscript:

MDPI	Multidisciplinary Digital Publishing Institute
DOAJ	Directory of open access journals
OSA	Obstructive sleep apnea
CPAP	Continuous positive airway pressure
ROS	Reactive oxygen species
AHI	Apnea-hypopnea index
AF	Atrial fibrillation
ERP	Effective myocardial refractory period
PABs	Premature atrial beats
hs-CRP	High sensitive C reactive protein
NO	Nitric oxide
NF-κB	Nuclear factor kappa light chain enhancer of activated B cells
DNA	Deoxyribonucleic acid
ADP	After depolarizing potentials
CI	Confidence interval
ANS	Autonomic nervous system
GP	Ganglionated plexi
RTX	Resiniferatoxin
CSA	Central sleep apnea
HFrEF	Heart failure with reduced ejection fraction
HFpEF	Heart failure with preserved ejection fraction
LTCC	L-type calcium channels
TRPC	Transient receptor potential canonical channels
TRPC1	Transient receptor potential canonical 1
TRPC6	Transient receptor potential canonical 6
APD	Action potential duration
SDB	Sleep disordered breathing
NABS	Neck Circumference, Age, Body Mass Index, Snoring
PSG	Polysomnography
HSAT	Home sleep apnea testing
SCD	Sudden cardiac death
HRV	Heart rate variability
BMI	Body Mass Index
TMAO	Trimethylamine N-oxide
STEMI	ST-elevation Myocardial Infarction
HNS	Hypoglossal nerve stimulation
DISE	Drug-induced sleep endoscopy
TORS	Transoral robotic surgery
RDI	Respirator Disturbance Index
ICD	Implantable cardioverter-defibrillator
UPPP	Uvulopalatopharingoplasty
CHF	Congestive heart failure
